# Exploring the Antioxidative Effects of Ginger and Cinnamon: A Comprehensive Review of Evidence and Molecular Mechanisms Involved in Polycystic Ovary Syndrome (PCOS) and Other Oxidative Stress-Related Disorders

**DOI:** 10.3390/antiox13040392

**Published:** 2024-03-25

**Authors:** Sladjana Novakovic, Vladimir Jakovljevic, Nikola Jovic, Kristina Andric, Milica Milinkovic, Teodora Anicic, Bozidar Pindovic, Elena Nikolaevna Kareva, Vladimir Petrovich Fisenko, Aleksandra Dimitrijevic, Jovana Joksimovic Jovic

**Affiliations:** 1 Faculty of Medical Sciences, University of Kragujevac, 34000 Kragujevac, Serbia; sladja.novakovic.sumarice@gmail.com (S.N.); teodora.anicic95@gmail.com (T.A.); alexandrad84@gmail.com (A.D.); 2 Department of Physiology, Faculty of Medical Sciences, University of Kragujevac, Svetozara Markovica 69, 34000 Kragujevac, Serbia; drvladakgbg@yahoo.com; 3 Center of Excellence for Redox Balance Research in Cardiovascular and Metabolic Disorders, 34000 Kragujevac, Serbia; 4 Department of Human Pathology, I.M. Sechenov First Moscow State Medical University, Moscow 119048, Russia; 5 Department of Gynecology and Obstetrics, Faculty of Medical Sciences, University of Kragujevac, Svetozara Markovica 69, 34000 Kragujevac, Serbia; docctorny@gmail.com; 6 Clinic for Gynecology and Obstertics, University Clinical Center Kragujevac, Zmaj Jovina 30, 34000 Kragujevac, Serbia; 7 Department of Dermatovenerology, Faculty of Medical Sciences, University of Kragujevac, Svetozara Markovica 69, 34000 Kragujevac, Serbia; kristinajoksimovic16.2016@gmail.com; 8 Center for Dermatology, University Clinical Center Kragujevac, Zmaj Jovina 30, 34000 Kragujevac, Serbia; 9 Department of Biochemistry, Faculty of Medical Sciences, University of Kragujevac, Svetozara Markovica 69, 34000 Kragujevac, Serbia; milicamilinkovic15@yahoo.com; 10 Department of Pharmacy, Faculty of Medical Sciences, University of Kragujevac, Svetozara Markovica 69, 34000 Kragujevac, Serbia; pindovic.bozidar@gmail.com; 11 Department of Pharmacology, I.M. Sechenov First Moscow State Medical University, Moscow 119048, Russia; kareva_e_n@staff.sechenov.ru (E.N.K.); fisenko_v_p@staff.sechenov.ru (V.P.F.); 12 Institute of Public Health Kragujevac, 34000 Kragujevac, Serbia

**Keywords:** polycystic ovary syndrome PCOS, phytotherapy, cinnamon, ginger, antioxidant

## Abstract

Oxidative stress represents the pathophysiological basis for most disorders, including reproductive issues. Polycystic ovary syndrome (PCOS) is heterogeneous endocrine disorder of women characterized primarily by irregular menstrual cycles, hyper-androgenism, and ovulatory dysfunction. In the last decades, PCOS was recognized as a systemic silent inflammation and an oxidative disturbance-related disorder, exerting multifaceted symptoms, including metabolic. PCOS treatment should involve a personalized approach tailored to individual symptoms; however, the results are often unsatisfactory. Various supplementary treatments have been proposed to assist in the management and alleviation of PCOS symptoms. Cinnamon and ginger, known for millennia as herbs used in spices or traditional medicine for the treatment of various diseases, are of interest in this study. The aim of this study is to evaluate and investigate the effects of cinnamon and ginger in PCOS patients. Using relevant keywords we searched through PubMed/MEDLINE, Science Direct, Google Scholar and Web of science to find animal studies, pre-clinical, and clinical studies which were then reviewed for usage. Out of all of the reviewed studies a total of 65 studies were included in this review article. Cinnamon and ginger can affect hormonal status, lipid profile, obesity, and insulin resistance by mitigating oxidative stress and inflammation. Generally, based on current clinical evidence, it was revealed that supplementation with cinnamon or ginger had a useful impact in patients with PCOS. This review summarizes the antioxidative effects of ginger and cinnamon in PCOS treatment, highlighting their potential benefits in other oxidative stress-related pathologies.

## 1. Introduction

Polycystic ovary syndrome (PCOS) is a heterogeneous endocrine disorder in women, first described by Stein and Leventhal in 1935 [1]. It manifests with irregular menstrual cycles, hyperandrogenism, and ovulatory dysfunction [2]. Epidemiological data suggest that one in every 5–6 females experiences various PCOS symptoms. The prevalence of PCOS varies worldwide, ranging from 4 to 21% or 9.1 to 36%, contingent upon diagnostic criteria. Although clinical features exhibit considerable variation among individuals, the primary symptoms include irregular periods, acne, hirsutism, weight gain or difficulty losing weight, mood swings, and infertility [1]. According to the Rotterdam criteria, the diagnosis of PCOS requires the presence of more than two criteria (polycystic ovaries confirmed by ultrasound, hyperandrogenism, and anovulation) [3].

The etiology of PCOS remains incompletely understood, but environmental and genetic factors contribute to its development. In patients with PCOS, an increased risk for comorbidities such as insulin resistance, obesity, type 2 diabetes (DMT2), and cardiovascular disease has been demonstrated [1].

The pathophysiological mechanisms for PCOS are complex and many of them are yet to be completely revealed. PCOS pathophysiology is primarily related to the irregular function of the hypothalamic–pituitary–ovarian axis, abnormalities within ovaries, and impairment of insulin action and secretion [4]. The hormonal imbalance caused by the existence of an alteration in the hypothalamus–pituitary–ovary axis is one of the factors involved in the onset of PCOS [5]. The majority of women with PCOS experience hyperandrogenism, chronic anovulation, and insulin resistance [6]. Understanding the molecular mechanisms underlying PCOS is crucial for finding a curative therapy. In PCOS, an elevated level of gonadotropin-releasing hormone (GnRH) leads to the hypersecretion of LH over FSH production. This increased LH/FSH ratio induces hyperandrogenism and ovulatory dysfunction. In women with PCOS, elevated levels of E1 (estrone) and E2 (estradiol) are associated with peripheral conversion of androgens and increased androgen- independent aromatase activity of granulosa ovary cells [7]. Estrogen decreases FSH levels and increases the LH/FSH ratio as a consequence of the negative FSH feedback mechanism. A high level of LH activates the ovary to produce androgens, while the low level of FSH prevents follicle growth and leads to anovulation [6]. Other researchers asserted that this is caused partially by the elevated level of LH and the LH/FSH ratio [7]. This occurrence is a characteristic in 80% of PCOS cases, and in obese women, the prevalence of PCOS is even higher. In addition to the obvious negative impact on the menstrual cycle, hyperandrogenism can also cause hirsutism, acne, and androgenic alopecia which are all serious problems for women with PCOS [5]. Additionally, women with PCOS exhibit higher blood insulin levels (hyperinsulinemia), which prematurely arrests follicle development by interacting with LH (luteinizing hormone) [2]. Insulin resistance, characterized by elevated insulin levels in the bloodstream and decreased cell responsiveness to insulin, affects not only overweight but also lean women [8]. It is also worth noting that insulin signaling is also involved in the complex pathophysiological mechanisms in PCOS. Insulin directly promotes androgen secretion via ovarian theca cells. In addition, insulin stimulates ovarian follicle development, playing a crucial role in facilitating steroid hormone production and oocyte maturation. On the other hand, insulin modulates LH pulse amplitude, suppresses hepatic SHBG production and inhibits IGF-1 binding protein production in the liver, which is responsible for triggering the secretion of androgens in thecal cells. Inhibition of the production of IGF-1 binding proteins leads to a higher concentration of this substance in blood circulation and therefore a higher production of androgens by thecal cells [6]. Under the influence of insulin, the ratio of follicle-stimulating hormone (FSH) and LH changes, and the level of LH increases significantly. LH hypersecretion stimulates the cells to produce androgens and reduces the effect of FSH [9]. 

In addition to the previously identified factors in the pathophysiology of PCOS, oxidative stress and inflammation have also been recognized as a molecular basis in PCOS onset. Increased levels of markers of oxidative stress activate the nuclear factor-kappa B (NF-κB) which is involved in inflammatory pathways and production pro-inflammatory cytokines like TNF-α and IL-6. In addition, increased oxidative stress activates some protein kinases that trigger serine/threonine phosphorylation instead of normal tyrosine phosphorylation of IRS (insulin receptor substrate). Insulin signaling pathway is inhibited and as a result of that process, insulin resistance occurs [8]. Chronic low-grade inflammation present in PCOS patients is associated with excess androgens, insulin resistance, and obesity. The levels of C-reactive protein, interleukins, and tumor necrosis factor- α are elevated, while SHBG levels are often lowered. This suggests that inflammatory cytokines may regulate SHBG expression, although the mechanism of this regulation has not been sufficiently understood [7]. The increase in the level of adipokines (leptin), cytokines (TNF-α and IL-6), and non-esterified fatty acids in obese patients can block the action of insulin and help develop insulin resistance, which leads to T2DM. Visceral obesity, as one of the serious problems of women with PCOS, has been shown to reduce plasma levels of SOD and GPx [10].

According to the 2023 International Evidence-based Guideline for the Assessment and Management of Polycystic Ovary Syndrome, oral contraceptive pills are the preferred initial medication for managing menstrual irregularity and excess androgens. Letrozole is the first-line of pharmacological infertility therapy, while clomiphene alone or in combination with metformin, followed by gonadotrophins or ovarian surgery, are fulfilling the role of second-line therapy. Metformin is primarily recommended for metabolic PCOS issues, while it is not routinely recommended for use in pregnant women with PCOS [11]. Despite this, there is growing interest in lifestyle modifications including diet, exercise, and traditional herbal remedies within global health conversations [12]. The side effects of the pharmacological treatments often lead to the cessation of therapy, while therapeutic resistance or low adherence could also be observed. Consequently, there is an urgent demand to explore and enhance the efficacy of plant-derived medications over conventional allopathic drugs. Several studies have explored the potential of alternative medicine in mitigating symptoms associated with PCOS [13]. Traditional medicine has been recognized for its promotional, preventative, curative, and rehabilitative roles. One advantage of herbal therapy compared to conventional treatments is its safety and fewer side effects. Additionally, medicinal herbs often contain multiple active compounds, leading to a synergistic effect [1]. Previous studies have revealed that numerous herbs contain pharmacologically active constituents that positively affect ovulatory dysfunction, obesity, insulin resistance, and hormonal status [14]. Cinnamon and ginger, traditionally recognized for their medicinal properties for millennia, have shown therapeutic effects on various aspects of PCOS.

Ginger, derived from the rhizomes of the plant *Zingiber officinale*, primarily grown in Asia and tropical areas, has been used for its medicinal properties and as a spice for thousands of years. Ginger rhizomes contain various bioactive compounds like phenols, terpenes, and volatile oils. The compound 6-gingerol provides its pungent taste and exhibits antioxidative and anti-inflammatory activity [15]. Cinnamon, from the genus *Cinnamonum,* comprising numerous species, has been used as a food, spice, or as an alternative remedy for years. Common species include *Cinnamomum verum*, *Cinnamomum cassia* (Chinese cinnamon), and *Cinnamomum zeylanicum* [16]. All cinnamon species contain cinnamaldehyde, eugenol, various minerals like manganese, iron, calcium, dietary fiber, and related compounds [17].

In this review, we tried to provide a comprehensive overview of the current understanding of beneficial effects exerted by ginger and cinnamon administration and molecular mechanism modulation in PCOS. Ensuring adherence to established guidelines and recommendations, we focused on alternative treatment options such as supplementation with ginger and/or cinnamon as an adjuvant in coping multifaceted PCOS features. Finally, we provided evidence from studies regarding the beneficial effect of these two herbs in different oxidative stress-related disorders excluding PCOS, emphasizing their potential as antioxidative agents.

## 2. Materials and Methods

The literature search for this review article was performed using the following databases: PubMed/MEDLINE, Science Direct, Google Scholar, and Web of Science.

We used a combination of a few keywords and free words (AND, OR): ‘polycystic ovary syndrome’, ‘PCOS’, ‘phytotherapy’, ‘cinnamon’ and ‘ginger’. 

The inclusion criteria in this study were articles in English language, animal studies, pre-clinical, and clinical studies with free full text published from 2003 to 2023. On the other side, review articles, systematic review articles, meta-analyses, case reports, short communications, and letters to editors were excluded from the study. We also excluded duplicated studies or studies from which data of interest could not be extracted. From the total number of studies, 65 were finally selected and used in writing this article.

## 3. Results

This review summarizes the studies conducted on humans and animal models (mice or rats) that showed the efficacy of cinnamon (see Table 1) and ginger (see Table 2) and their active principles in treating PCOS. Most of the studies showed the beneficial effects of these herbs on the lipid profile, serum levels of sex hormones, glucose levels, insulin action, and redox status.

Cinnamon species have been investigated in clinical and animal studies as a form of treatment for PCOS. It was demonstrated that cinnamon administered as powder restored estrous cyclicity, decreased the levels of testosterone and insulin, and increased the levels of LH in DHEA-induced PCOS mouse models [18]. In another study, rats were given aqueous extract of cinnamon bark for 15 days. Results indicate that supplementation with cinnamon increases the levels of progesterone, estradiol, LH, and FSH [19]. In line with those findings, a study by Khaki showed that cinnamon extract regulated oxidative damage and improved biochemical indices in PCOS rats. In Wistar female rats, plasma levels of GPx, SOD, and MDA have been decreased after cinnamon supplementation [20]. Antioxidant compounds in cinnamon protect ovarian tissue from oxidative stress by decreasing lipid peroxidation, superoxide dismutase activity, glutathione peroxidase activity, and increasing total glutathione levels in the cells [14].

In addition to the numerous studies that examined the effects of cinnamon in animal models of PCOS, there are a few studies conducted on PCOS patients with various findings. A clinical trial conducted by Borzoei et al. on 84 overweight or obese women with PCOS diagnosis showed that treatment with 1500 mg cinnamon per day decreased the levels of triglycerides (TG), serum fasting blood glucose levels, and HOMA-IR. Polyphenols isolated from cinnamon increase insulin dependent glucose metabolism by activating the insulin receptor and altering glucose transport. In addition, cinnamon has established homeostatic model assessment for insulin resistance [21]. In another study, authors revealed similar effects. Serum levels of total cholesterol and low-density lipoprotein cholesterol were decreased. Treating women with cinnamon improved antioxidant status by increasing TAC levels and increasing MDA levels [22]. A double-blind controlled trial conducted by Hajimonfarednejad et al. revealed that administrating 1.5 g/day of cinnamon divided in three doses can significantly reduce total cholesterol levels. Fasting insulin and homeostatic model assessment for insulin resistance were reduced after 12 weeks of cinnamon intake [23]. Cinnamon supplementation in 45 women with PCOS showed opposite results compared to previous studies. After taking 75 g of glucose orally, tolerance test results showed that insulin resistance did not significantly change over the study period in either group. In addition, the waist circumference and serum androgen levels in women did not significantly change; however, menstrual cyclicity was regulated in women taking cinnamon compared to placebo [24].

Sixty-three PCOS-rats were divided into groups and treated with 100 mg/kg of clomiphene citrate or ginger extract in different doses (175 and 350 mg/kg) orally for 60 and 89 days. Plasma levels of LH and estrogen were increased, while progesterone and FSH levels decreased in PCOS-induced groups as compared to the control group. In groups receiving clomiphene or ginger extract, a significant improvement in secretion of hormones as compared to the PCOS-induced group was demonstrated. However, better results were registered in rats receiving clomiphene citrate than ginger extract [25]. Moreover, the dose-dependent effects of ginger extract were observed in the mentioned study. Higher doses exerted stronger effects in improving hormonal status after PCOS induction. Ginger extract contains gingerols and sesquiterpens which inhibit prostaglandin production by inhibiting arachidonic acid production [15]. These findings are similar to another study where PCOS rats showed a significant decrease in serum levels of LH, FSH, estradiol, and testosterone as compared to a PCOS control group, but levels of progesterone were increased, which is in opposite to the previous study [26]. Pouranderi et al. revealed that 6-gingerol is more effective than ginger extract in improving PCOS through reduction in testosterone, estrogen, LH, FSH, and the presence of ovarian cysts. Treating PCOS rats with 6-gingerol in dose 400 µg/kg showed a significant decrease in FSH levels [27]. Results from this study are partially in agreement with another study where 6-gingerol administered to rats showed an increase in all mentioned hormones, except progesterone levels which were decreased in the PCOS group compared to those in control [28].

The supplementation of women with PCOS with ginger capsules in a dose of 3 g per day for 12 weeks showed a decrease in levels of LH, testosterone, and insulin levels, especially when combined with pilates exercises. There were no significant differences between the exercise group and the ginger supplementation group, except in the measured weight of women, where the exercise group showed better results [29]. Results of the study conducted by Alghamdi confirmed a previous finding, where women treated with ginger capsules had lower values of BMI, insulin, and high and low-density lipoproteins [30].

### 3.1. The Effects of Ginger and Cinnamon on Sex Hormones

PCOS is a disease characterized by a disproportion of reproductive hormones which affect the normal menstrual cycle and lead to anovulation and infertility in women. Women with this syndrome often exhibit an increased frequency of GnRH pulsations, which further elevates the secretion of LH and FSH. In most cases, the serum level of testosterone, estradiol, and LH is elevated while the levels of progesterone and FSH are decreased. In some cases, there is no change in the serum level of FSH [2].

Previous studies on PCOS mice models induced by DHEA indicated that the administration of cinnamon powder at a dose of 10 mg/100 g body weight could decrease the serum level of testosterone, FSH, insulin, IGF-1, and IGFBP-1, while LH levels could increase compared to the control group. Active components of cinnamon like quertin, catechin, and rutin, contribute to the improvement in insulin sensitivity. In fact, cinnamon acts like an insulin, or like an enhancer of insulin receptor function. The synthesis of androgens in an ovary act through IGF-1 receptors on a granulosa cell of small antral follicles under the influence of insulin. High levels of insulin lead to high levels of IGF-1 and androgens, resulting in anovulation [18]. Similar results were clinically confirmed by Kort DH et al. in a study on women who orally consumed four cinnamon capsules three times a day (each 125 mg cinnamon) [21]. 

In one study with rats with induced PCOS where they were administered *Cinnamon zeylanicum* (CZ) extract orally (200 mg/kg) for 14 days, results showed that the serum level of LH and testosterone were decreased and the level of FSH was increased compared to the control [24]. Other authors also conducted experiments on rats with induced PCOS. They treated rats with an aqueous extract of *Cinnamon zeylanicum* at a concentration of 5 mg/mL per os for a period of 15 days. Levels of progesterone, estradiol, LH, and FSH were increased significantly compared to the control [22]. Ginger supplementation (500 mg of ginger, 3 × day) in a study with 100 women with PCOS showed significant decreases in FSH (follicle-stimulating hormone) and LH (Luteinizing hormone) levels compared to the placebo, while a cinnamon supplementation of 500 mg 3 × day lowered testosterone levels [31].

### 3.2. The Effects of Ginger and Cinnamon on Obesity and Overweight

Many women with PCOS experience problems with obesity, although PCOS also occurs in lean women. PCOS women with obesity usually have increased values in fasting glucose, lipid profile, and have insulin resistance [2]. Obesity induces mild-chronic inflammation in the adipose tissue which increases insulin resistance. Samera A., in his study, showed that ginger reduces serum levels of fasting glucose, glucose, and low- and high-density lipoproteins [30]. 

A few authors demonstrated that HED-induced rats treated with gingerol orally have lower levels of body weight, glucose, lipid profile, leptin, and amylase. The best effect was observed with a dose of 75 mg/kg body weight [32]. In a study with rats who were on a high-fat diet (HFD), authors showed that an ethanolic extract of *Z. officinale* reduced body weights, total cholesterol, LDL cholesterol, and triglycerides [33]. In another study, authors revealed that rats treated with HFD containing gingerol had lower weight gain, fat accumulation, and circulating levels of leptin compared to HFD control [32,34,35]. These findings are consistent with some earlier studies, where intake of ginger aqueous extract significantly decreased body weight, body fat mass and serum leptin levels in obese diabetic rats compared to the control group [36]. Contrary to this study, another study showed that ginger juice significantly increased weight gain and decreased leptin levels in rats [37]. 

Study conducted on mice who were exposed to HFD demonstrated that coadministration of ginger extract in a dose of 500 mg/kg (*w*/*w*) corrected the HFD-induced increases in body weight, fat accumulation, and levels of serum glucose, triglyceride, and cholesterol. Ginger prevents obesity by activating white adipose tissue browning by altering the gene expression and protein levels of some brown and beige adipocyte-selective markers [38].

In a double-blind randomized study on obese women who received 2 g/day of ginger powder for 12 weeks, a reduction in BMI and serum insulin was shown, along with no significant reductions in serum leptin, resistin, and glucose as compared to the placebo group [39]. These findings are partly in agreement with other experimental studies. Mansour et al. demonstrated that the consumption of a single-dose hot ginger beverage (2 g ginger powder per day) before a standard breakfast meal decreased hunger and prospective food intake but had no significant effect on serum leptin, adiponectin, and other cytokines levels, as compared to the control group [40].

Cinnamon also has anti-obesity effects. Orally administered cinnamon in PCOS women reduced the levels of total cholesterol, triacylglycerol, and high-density lipoprotein cholesterol [19,24]. Vafa et al. also showed that supplementation with a cinnamon dose of 3 g a day for 8 weeks decreased the body weight of subjects with type 2 diabetes [41]. 

A randomized controlled trial conducted on Asian Indians with metabolic syndrome showed a significant decrease in hyperglycemia, body weight, total adiposity, abdominal adiposity and serum lipid levels using 3 g/day of cinnamon compared to placebo over a period of 16 weeks. A detail that sets this study apart is a significant increase in serum HDL-C levels upon cinnamon intake [42].

**Figure 1:**
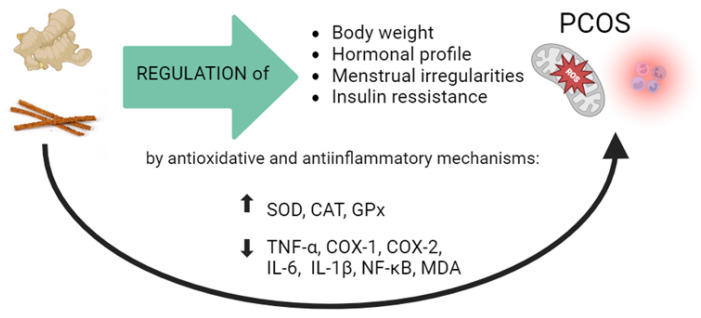
The beneficial effects of ginger and cinnamon in mitigating PCOS-related symptoms by modulation of molecular mechanisms involved in oxidative stress and inflammation.

In a study with diabetic patients, Khan et al. found that using cinnamon in three different doses (1, 3, and 6 g/day) for a period of 40 days reduced the levels of total serum cholesterol, triglycerides, LDL-C [43]. One more study confirms these findings, where 22 subjects with prediabetes and metabolic syndrome consumed 500 mg/day of a specific aqueous extract of cinnamon for 12 weeks. Results indicated a significant improvement in fasting blood sugar, systolic blood pressure, and body composition [44]. We present you a figure summarizing some of the beneficial effects of ginger and cinnamon in mitigating PCOS-related symptoms (Figure 1).

Regarding the factors associated with PCOS mentioned in the manuscript (insulin resistance, hormonal imbalance, lipid profile abnormalities), while there is a strong correlation between these factors and PCOS, establishing causation is complex and multifactorial. PCOS is a heterogeneous disorder with various contributing factors, including genetic predisposition, environmental influences, and metabolic dysregulation. Insulin resistance, for example, is commonly observed in individuals with PCOS, but it is unclear whether it precedes or follows the development of PCOS. Similarly, hormonal imbalances involving gonadotropins, androgens, and estrogens are characteristic features of PCOS, but their causal relationship with the syndrome remains to be fully elucidated.

### 3.3. Molecular Mechanisms Modulation by Ginger and Cinnamon on PCOS-Related Oxidative Stress and Inflammation

Oxidative stress represents the pathophysiological basis for most disorders, including reproductive disorders. In the last decades, PCOS was recognized as a systemic silent inflammation and an oxidative disturbance-related disorder (Figure 1). Oxidative stress arises from an imbalance between oxidant generation and antioxidant defenses within cells. Elevated levels of reactive oxygen species (ROS) in PCOS lead to oocyte damage, suggesting that reducing oxidant markers could represent a potential therapeutic mechanism offered by numerous herbs. Key markers of oxidative stress include various ROS, MDA (malondialdehyde), and HCy (homocysteine), while markers of antioxidant capacity encompass systems of enzymatic and non-enzymatic defense, where the most important factors were recognized as the following: SOD (superoxide dismutase), CAT (catalase), and glutathione cycle components [45].

In addition to insulin resistance and hormone imbalance, chronic low-grade inflammation is one of the several factors which contribute to the pathogenesis of PCOS. In PCOS, the expression of tumor necrosis factor (TNF-α), COX-2, IL-6, and IL-8 is shown to be increased [46]. Pouranderu, Yaghamei et al. have shown that the active component in ginger, 6-gingerol, reduces mRNA levels of COX-2 in a PCOS group of rats [29]. Similar to previous findings, another study also confirmed that the administration of 6-gingerol decreased COX-2 expression in ovarian tissue of rats with induced PCOS [27].

A study conducted on mice that underwent a high-fat diet and were administered cinnamaldehyde, a bioactive component of cinnamon, showed that cinnamaldehyde administered in the form of an intraperitoneal injection (10 mg/kg) decreased serum IL-1β levels in visceral white adipose tissue [47]. Another study investigated the effect of cinnamaldehyde as a form of treatment for rats with allergic rhinitis. Results showed that cinnamaldehyde decreased vascular congestion as well as plasma cell, eosinophil, and inflammatory cell infiltration into the lamina propria [48]. Oral administration of aqueous *C. burmannii* extract to rats who were exposed to multi-walled carbon nanotube showed a reduction in the rate of pro-inflammatory cytokines including interleukin-6 (IL-6), interleukin-1β (IL-1β), cyclooxygenase-1 (COX-1), and tumor necrotic factor-α [49].

The beneficial effects of ginger in PCOS are thought to be mediated through various molecular mechanisms. Ginger contains bioactive compounds like gingerols, shogaols, and gingerdione which have antioxidant and anti-inflammatory properties. These compounds suppress the gene expression of pro-inflammatory cytokines (TNF-α, IL-1β and IL-6) by inhibiting the activation of NF-κB signaling pathway [50]. Other authors suggested that ginger-derived nano particles can prevent insulin resistance in high-fat diet fed mice by increasing the expression of Foxa2 and protecting Foxa2 from AKT-1 mediated phosphorylation, ensuring inactivation of Foxa2 [51]. In another study, ginger extract showed insulin sensitizing effects in HFD rats. Polyphenols from ginger increase expression of PPARγ in liver and lead to enhanced GLUT-2 expression thus improving glucose transport and establishing glucose homeostasis [52]. Aqueous extract of ginger achieves anti-inflammatory activity through inhibition macrophage and neutrophils activation by suppressing production of IL1-β from macrophages [53]. Gingerol is known as the major bioactive component with antioxidative effect. Gingerol can stimulate the AMPK/NF-κB pathway in adipocytes to suppress proinflammatory signaling and block insulin resistance [54]. Ginger extract directly scavenges ROS, increases the endogenous antioxidant defense, and also reduces mitochondrial oxidative stress by decreasing the ccf-mtDNA levels in the plasma [55].

Cinnamon exerts beneficial effects in PCOS through various molecular mechanisms.

Cinnamon increases glucose uptake in muscles and adipose tissues by upregulating the expression of GLUT 4. Cinnamon also inhibits the activity of retinol-binding protein 4 (RBP4), an adipokine which is inversely correlated with the expression of the main GLUT4 transporter [56]. Data from previous studies suggests that cinnamon improves glucose metabolism disorders in two ways. The first way is by upregulating the IRS/PI3K/AKT2 signaling pathway. Cinnamon regulates phosphorylation of AKT (serine/threonine protein kinase) by activating PI3K, thereby inhibiting glycogen synthase kinase3β (GSK3β) and increasing glycogen synthesis. The second way is by regulating AMPKα/PGC1α-mediated hepatic gluconeogenesis [57]. Improvement of abnormal glucose tolerance can be also achieved through reducing ACSL1(Acyl-CoA synthetase long-chain family 1) expression in adipocytes and decreased fatty acid uptake into cells by inhibiting triacilglycerol synthesis. When energy sources are wasted, GLUT 4 will translocate to the plasma membrane [58].

Cinnamon and its compounds can regulate lipid profiles, but the mechanism of action is not clearly understood. Some researchers have found that cinnamon inactivates the Nieamann-Pick c1-like and Cd36 mRNA receptors on the enterocytes, leading to a decrease in absorption of free cholesterol and free fat acid [59].

### 3.4. The Antioxidative Potential of Ginger and Cinnamon: Evidence from Different Oxidative-Stress-Related Disorders excluding PCOS

Ginger and cinnamon, rich in antioxidant compounds, have demonstrated potential in preventing oxidative stress across various organ systems beyond PCOS, including the male reproductive, nervous, and gastrointestinal systems. Selected studies show the antioxidant potential of ginger and cinnamon from different oxidative stress-related disorders, excluding PCOS (see Tables S1–S7).

Morakinyo et al. conducted a study exposing rats to sodium arsenite to induce testicular toxicity, with one group receiving aqueous ginger extract at 500 mg/kg body weight for 30 days. Results indicated a decrease in MDA levels and an increase in GSH, SOD, and CAT levels [60], consistent with Ismail T et al.’s findings of increased antioxidant enzyme levels with Zingiber extract administration in rats with formalin-induced testicular toxicity [61]. Moreover, ginger improved sperm motility and viability [57].

Studies have also highlighted ginger’s protective effects on the male reproductive system in diabetic rats, with significant improvements in MDA levels and antioxidant enzymes (SOD, CAT, GPx) observed in the testis, epididymis, and prostate [62]. Furthermore, a study suggested a synergistic effect of ginger and cinnamon on spermatogenesis in diabetic rats compared to ginger alone [63].

Akbari et al. evaluated the prophylactic antioxidant effects of ginger extract on ethanol-induced reproductive toxicity in male rats, finding that pretreatment with ginger rhizome extract at 1 g/kg body weight increased SOD, CAT, and GPx levels and reduced MDA and tHCy levels [64].

In their study, Kulkarni and Deshpande explored the antioxidant effects of ginger in tuberculosis patients. They observed higher serum MDA concentrations in tuberculosis patients compared to the control group, indicative of increased lipid peroxidation. However, supplementation with 3 g of ginger daily for one month alongside antitubercular treatment led to a significant decrease in MDA concentration compared to the control group [65].

Peritoneal dialysis (PD) patients often experience complications due to decreased clearance of oxidant compounds resulting from kidney failure. In a study by Imani H et al., 36 PD patients were randomly assigned to receive either ginger or a placebo. After 10 weeks of ginger administration at a dose of 1000 mg/day, there was no significant change in serum MDA concentration compared to the placebo group, indicating that ginger had no effect on oxidative stress in PD patients [66]. However, contrasting findings were observed in a study by Reddy et al., where supplementation with ginger extract improved renal toxicity induced by lead exposure in male rats. The administration of ginger extract increased levels of antioxidant enzymes (GSH, GPx, GST, CAT) and ameliorated histopathological changes induced by lead in the kidneys [67].

Oxidative stress plays a significant role in the pathogenesis of diabetes complications such as nephropathy. Afshari et al. induced diabetes in rats with streptozotocin and treated them with ginger extract. Ginger treatment significantly reduced TBARS levels and attenuated vascular thickening, glomerular proliferation, and hyalinization [68]. Similar antioxidant effects were observed with cinnamon in a study by Mishra et al., where cinnamon oil administration reduced TBARS levels in a dose-dependent manner and increased GSH levels, with the highest response observed at a dose of 20 mg/kg of body weight [69]. These findings were corroborated by a study by Alshahrani S et al., where cinnamon oil administration increased antioxidant enzyme levels and decreased lipid peroxidation in rats with kidney damage induced by acetaminophen [70].

Additionally, Morgan et al. investigated the antioxidative effect of cinnamon aqueous extract against oxidative stress induced by bisphenol-A (BPA) and octylphenol (OP). Pretreatment with cinnamon extract mitigated adverse effects induced by BPA and OP exposure, including changes in body weight, organ weight, and biochemical parameters [71]. Similarly, Sakr and Albarakai found that cinnamon improved kidney injury induced by the pyrethroid insecticide cypermethrin in rats, leading to histological improvements and increased antioxidant enzyme levels [72].

Sharma and Singh conducted a study to assess the neuroprotective effect of ginger against neurotoxicity induced by lindane and dichlorvos in Wistar rats. These pesticides damage the lipoidal matrix in cells and induce oxidative stress, particularly in cerebral tissue. Administering ginger juice at a dose of 100 mg/kg for 14 days reduced lipid peroxidation levels and increased the levels of antioxidant enzymes such as CAT, SOD, GPx, and GST in rats exposed to lindane and dichlorvos [73].

Shaumugam et al. investigated the effect of ginger on oxidative stress parameters in streptozocin-induced diabetic rats. Ginger decreased the levels of malondialdehyde (MDA) in the mitochondrial fractions of the cerebral cortex, cerebellum, hippocampus, and hypothalamus, which were elevated in diabetic rats [74].

Ashafaq et al. explored the antioxidant role of cinnamon in male albino rats overdosed with acetaminophen, which induced neurotoxicity. Acetaminophen in high doses accumulates in the brain and induces oxidative stress in cortical neurons. Administration of cinnamon oil alongside acetaminophen increased the levels of antioxidant enzymes (GPx, GR, SOD, and CAT) in brain tissue. Moreover, the addition of cinnamon oil reduced lipid peroxidation levels [75].

Modi et al. investigated the therapeutic potential of cinnamon and its metabolite sodium benzoate in Alzheimer’s disease. Oral administration of cinnamon powder produced sodium benzoate in the hippocampi of mice, reducing the production of reactive oxygen species (ROS) and protecting memory and learning in an animal model of Alzheimer’s disease [76].

Taha et al. studied the antioxidant effect of cinnamon and ginger on the prefrontal cortex of nicotine-exposed rats. Nicotine increased MDA levels and decreased GSH levels in the prefrontal cortex compared to the control group. Administration of cinnamon (at a dose of 400 mg/kg) and ginger oil (at a dose of 50 mg/kg) separately, but particularly together, exhibited synergistic antioxidant and anti-inflammatory effects on the prefrontal cortex injured by nicotine [77].

Oxidative stress plays a crucial role in ulcerative colitis, particularly in its initiation and the occurrence of relapses. In this chronic inflammatory disorder, the immune system is compromised, leading to the production of reactive oxygen species (ROS) that damage the integrity of the intestinal mucosa. Total antioxidant capacity (TAC) and malondialdehyde (MDA) levels, as indicators of oxidative stress, were assessed. Consumption of 2000 mg/day ginger significantly decreased serum MDA levels compared to the control group after 6 and 12 weeks, while no significant difference was observed in TAC levels between the groups. Moreover, ginger supplementation significantly improved patients’ quality of life at the 12-week mark [78].

Attia et al. exposed rats to lead acetate for 50 days to induce hepatic injury, resulting in increased plasma levels of ALT, AST, and ALP, as well as elevated MDA levels and decreased glutathione (GSH) concentrations in liver tissue homogenate. Administration of aqueous ginger solution orally at a dose of 160 mg/kg attenuated oxidative stress by decreasing MDA levels and increasing GSH and antioxidant enzyme (GPx and SOD) levels in the livers of lead-treated rats [79].

Danwilai et al. examined the antioxidant effect of ginger extract administered orally to cancer patients receiving adjuvant chemotherapy compared to placebo. After 64 days of ginger administration, there was an increase in antioxidant enzyme levels (CuZn-SOD and CAT activity) and a decrease in oxidative stress markers (MDA and NO_2_^−^/NO_3_^−^) compared to the placebo [80].

Tuzcu et al. conducted a study in which Wistar rats were fed a high-fat diet (HFD) for 12 weeks to induce obesity. Treatment with cinnamon polyphenol extract at 100 mg/kg reduced serum and liver MDA concentrations and increased serum total antioxidant capacity (TAC), as well as liver SOD, CAT, and GPx levels, similar to those in the control group [81].

Haidari et al. investigated the effect of cinnamon extract on acrylamide (AA)-induced toxicity in rats. Cinnamon extract administration decreased MDA levels and increased TAC levels in a dose-dependent manner, while also restoring aminotransferase levels [82].

Hussain et al. studied the effect of cinnamon oil on acetaminophen-induced liver toxicity in rats. Administration of cinnamon oil restored the balance between oxidant and antioxidant parameters, which had been disrupted by high doses of acetaminophen [83].

Moselhy and Ali investigated the hepatoprotective effect of cinnamon against carbon tetrachloride-induced liver toxicity in rats. Administration of ethanolic cinnamon extract lowered MDA levels and increased antioxidant enzyme (SOD and CAT) levels, while also restoring hepatic enzymes (ALT and AST) [84].

Amin et al. examined the effect of cinnamon and atorvastatin on serum lipid and antioxidant capacity in hypercholesterolemic rats. Cinnamon demonstrated similar effects to atorvastatin on lipid status and enhanced nitric oxide levels without causing side effects [85].

Furthermore, cinnamon decoction reduced blood glucose levels and improved antioxidant enzyme levels in the liver, as well as elevating liver marker enzymes in serum in rats [86]. Additionally, cinnamon decoction exhibited a protective effect against gamma radiation-induced oxidative stress [87].

Amoxicillin clavulanate (AC) administration in rats caused dose-dependent hepatocellular injury, marked by increased liver enzyme activities and levels of MDA, hydrogen peroxide (H_2_O_2_), and nitric oxide (NO). Cinnamon administration reduced MDA, H_2_O_2_, and NO levels and improved liver enzyme activities, along with increasing antioxidant parameters [88].

Atashak confirmed that ten weeks of progressive resistance training can enhance antioxidant defense capacity and reduce malondialdehyde (MDA) and total antioxidant capacity (TAC) levels in obese men, with similar effects observed with ginger supplementation. However, when training and supplementation were combined, the effects were nullified [89].

A human study conducted on 22 overweight or obese subjects found that supplementation with aqueous cinnamon extract capsules (250 mg/kg) twice daily for 12 weeks decreased plasma MDA levels and increased Ferric Reducing Activity of Plasma (FRAP) and plasma SH groups, indicating improved antioxidant status. However, supplementation did not alter superoxide dismutase (SOD) and glutathione peroxidase (GPx) levels. Additionally, a positive correlation between MDA levels and plasma glucose was noted, suggesting that the antioxidant effects of cinnamon extract are associated with the improvement of impaired fasting glycemia [90].

In another study, patients with type 2 diabetes mellitus (DM) received cinnamon capsules (500 mg) or placebo daily for 60 days. Supplementation with cinnamon reduced MDA levels and increased TAC, whereas the placebo group showed a significant increase in MDA and a decrease in TAC [91].

Mashaddi et al. investigated the effects of cinnamon and ginger supplementation on oxidative stress and exercise performance in female taekwondo players. Forty-nine healthy women received oral dietary ginger or cinnamon powder (3 g) or placebo for 8 weeks. While there was a minor, nonsignificant decrease in MDA levels in the cinnamon and ginger groups compared to the placebo, cinnamon supplementation resulted in increased skinfold thickness and improved exercise performance in the ginger group [92].

Khandouzi et al. conducted a study to investigate the effect of ginger on various parameters, including malondialdehyde (MDA), in patients with type 2 diabetes. Patients were randomly assigned to receive ginger powder supplements (2 g/day) or placebo. Ginger supplementation significantly reduced levels of glycated hemoglobin (HbA1c) and MDA, and increased levels of apolipoprotein A-I (Apo A-I) in type 2 diabetic patients [93].

An animal study revealed the protective effect of ginger on tissue antioxidant defense systems in streptozocin-induced diabetic rats. Ginger supplementation for 30 days resulted in dose-dependent hypoglycemic and antioxidant activities, increasing levels of superoxide dismutase (SOD), catalase (CAT), glutathione peroxidase (GPx), glutathione reductase (GR), and glutathione (GSH), while reducing MDA levels [94].

Similar results were obtained in another study involving lindane-induced oxidative stress in rats. Dietary supplementation with ginger significantly attenuated lindane-induced lipid peroxidation and enhanced antioxidant enzyme activities, including SOD, CAT, GPx, GR, and GST [95].

In a study assessing the effects of supplementation with ginger, Gelam honey, and their combination in streptozocin-induced diabetic rats, three weeks of supplementation reduced levels of SOD, CAT, and MDA compared to the control group [96].

Morakynjo’s study on streptozocin-induced diabetic rats receiving different doses of aqueous and ethanol ginger extracts showed increased levels of SOD, CAT, and GSH after 6 weeks of treatment, confirming the antioxidant effects of ginger extract observed in previous studies [97].

Research on male diabetic rats investigated the effect of cinnamon on oxidative stress parameters. Administration of cinnamon powder at a dose of 75 mg/kg for 30 days significantly increased serum levels of SOD, CAT, GPx, and decreased MDA levels in all diabetic groups receiving cinnamon [98]. These findings were consistent with two other studies, which showed that cinnamon extract administration increased SOD and GPx enzyme activity, with the most significant effect observed at a dose of 200 mg/kg [99,100].

Administration of *Cinnamomum zeylanicum* extract in rats led to a reduction in infarct size, accompanied by significant elevations in serum superoxide dismutase and glutathione peroxidase activities, as well as a notable decrease in serum cardiac troponin I, lactate dehydrogenase, and malondialdehyde levels, observed five days after reperfusion. The most favorable outcomes were achieved at a dose of 200 mg/kg body weight [101].

A similar study was conducted on rats fed a high-fat, high-fructose diet (HFHFD), which resulted in signs of cardiomyopathy, including a significant increase in blood glucose, serum cardiac enzymes, inflammatory cytokines, and oxidative stress markers. Oxidative stress was evidenced by a significant decrease in serum antioxidant enzymes glutathione peroxidase (GPx) and total antioxidant capacity (TAC), along with an increase in malondialdehyde (MDA) levels. Administration of cinnamon aqueous extract at a dose of 200 mg/kg improved GPx and TAC levels and decreased MDA levels, with the best results observed when administered concurrently with pioglitazone [102].

## 4. Conclusions

Based on existing clinical evidence and preclinical studies, the supplementation of cinnamon or ginger has demonstrated significant beneficial effects in regulating serum levels of glucose, insulin, lipid profiles, sex hormones, oxidative stress markers, and inflammation in PCOS. While PCOS has been recognized as a multifaceted and heterogeneous disorder, the robust antioxidant properties inherent in ginger and cinnamon, primarily attributed to their phenolic constituents’ capacity to neutralize reactive oxygen species, are believed to underpin these protective attributes across multiple organ systems. Additionally, preclinical studies on animals and investigations into oxidative stress-related pathologies provided further insights into the potential mechanisms of action of ginger and cinnamon. These studies highlight the broader implications of these herbal remedies beyond PCOS, shedding light on their effects on various physiological pathways associated with oxidative stress and inflammation. However, notable variations in efficacy at different dosage levels underscore the necessity for comprehensive clinical studies employing larger sample sizes. Such investigations are imperative to thoroughly probe and ascertain the safety and efficacy profiles of these potential herbal remedies within the context of PCOS management, as well as to elucidate their broader therapeutic potential across a spectrum of oxidative stress-related conditions. In conclusion, while the clinical evidence supports the beneficial effects of cinnamon and ginger supplementation in PCOS management, further research integrating clinical and preclinical data is warranted to fully elucidate their therapeutic mechanisms and optimize their use in clinical practice.

## Figures and Tables

**Table 1:** Animal and clinical studies on the effect of cinnamon in treatment of PCOS.

Cinnamon					
Form of Drug	Reference	Name of the Study	Sample Size	Duration of Study/Doses	Parameters of Interest-Results
Cinnamon powder	[18]	The effect of cinnamon on polycystic ovary syndrome in a mouse model	60 female mice	Cinnamon powder 10 mg/100 g body weight in 100 µL 0.5% methylcellulose via gavage for 20 days	Testosterone, insulin, FSH ↓ LH ↑
Aqueous extract cinnamon bark	[19]	The Effect of Aqueous Extract *Cinnamon zeylanicum* Bark on the Structure and Function of the Ovary in Female Rats	20 female rats	Aqueous extract cinnamon bark of concentration 5 mg/mL for a period of 15 days	LH, FSH, Estradiol, Progesterone ↑
*Cinnamon zeylanicum* extract	[20]	The Effect of Hydroalchoholic Extract of *Cinnamon zeylanicum* on Oxidative Damages and Biochemical Change in Adult Rats With Polycystic	32 Wistar female rats	*Cinnamon zeylanicum* (CZ) extract orally (200 mg/kg) for 14 days	Plasma levels of GPX, SOD, MDA ↓ LH, testosterone ↓ FSH, Estrogen ↑
Cinnamon capsules (500 mg cinnamon powder)	[21]	Cinnamon improves metabolic factors without detectable effects on adiponectin in women with polycystic ovary syndrome;	84 women	3 times a day 1 capsule cinnamon powder for 8 weeks	Glucose Insulin TC, TG ↓ LDL-C HDL-C ↑
Cinnamon capsules (500 mg cinnamon powder)	[22]	Effects of cinnamon supplementation on antioxidant status and serum lipids in women with polycystic ovary syndrome	84 women	3 times a day 1 capsule cinnamon powder for 8 weeks	TAC levels ↑ MDA levels↓
Cinnamon capsules (500 mg cinnamon powder)	[23]	Insulin resistance improvement by cinnamon powder in polycystic ovary syndrome: A randomized double-blind placebo controlled clinical trial	59 women	3 times per day 500 mg cinnamon capsules or placebo for a period of 12 weeks.	Insulin LDL HDL Weight Triglyceride Testosterone ↓
Cinnamon capsules	[24]	Preliminary evidence that cinnamon improves menstrual cyclicity in women with polycystic ovary syndrome: a randomized controlled trial	45 women	3 × 4 capsules (125 mg) = 1500 mg a day or placebo capsules (4 capsules, 3×/day) for the 6 month study period.	Serum LH, testosterone insulin ↓ serum FSH levels ↑

↓—Decrease; ↑—Increase; IP—intraperitoneal; FSH—follicle stimulating hormone; LH—luteinizing hormone; TC—total cholesterol; TG—trygliceride; LDL—C low-density lipoprotein; HDL—C high-density lipoprotein; IGF-1—insulin-like growth factor; and IGFBP-1—insulin-like growth factor binding protein.

**Table 2:** Animal and clinical studies on the effect of ginger in treatment of PCOS.

Ginger					
Form of Drug	Reference	Name of the Study	Sample Size	Duration of Study/Doses	Parameters of Interest
Ginger extract	[25]	Comparison of the effects of Ginger extract with clomiphene citrate on sex hormones in rats with polycystic ovarian syndrome	63 adult female Wistar rats	I group: ginger extract (175 mg/kg/day) orally daily for 88 days. II group: ginger extract (350 mg/kg/day) orally daily for 88 days.	LH and estrogen serum level ↑ FSH and progesterone ↓
Ginger extract	[26]	The effects of ginger extract on cyclooxygenase-2 gene expression in polycystic ovary syndrome rats	35 adult female Wistar rats	I group: IP injection of 150 mg/kg ginger extract for 40 days. II group: IP injection of 300 mg/kg ginger extract for 40 days.	Estradiol ↓, Testosterone ↓, Progesterone ↑, FSH ↓ LH ↓
Ginger extract	[27]	Comparing the therapeutic effects of 6-gingerol and hydro-alcoholic extract of ginger on polycystic ovary syndrome in Wistar rats	42 adult female Wistar rats	(1) IP inj. of 100 mg/kg of ginger extract (for 28 days); (2) IP inj. of 200 mg/kg of ginger extract (for 28 days); (3) IP inj. of 200 μg/kg of 6-gingerol (for 14 days); (4) PCOS received IP inj. of 400 μg/kg of 6-gingerol (for 14 days	Estradiol ↓, progesterone ↑, testosterone ↓, LH and FSH ↓
6-gingerol solution	[28]	The effects of 6-Gingerol on reproductive improvement, liver functioning and Cyclooxygenase-2 gene expression in estradiol valerate-induced polycystic ovary syndrome in Wistar rats	36 adult female Wistar rats	Group G200: PCOS received IP inj. of 200 µg/kg 6-gingerol for 14 days Group G400: PCOS received IP inj. of 400 µg/kg 6-gingerol for 14 days.	FSH, LH, estradiol ↑ SOD, GPx ↓
Ginger capsules (1000 mg ginger powder)	[29]	The Effect of 12-week Pilates Training and Ginger Supplementation on Polycystic Ovary Syndrome in Women	40 women	The Ginger supplement group consumed daily 3 g of Ginger powder (poured in capsules) for 12 weeks with main meals	LH, testosterone, insulin levels ↓ FSH ↑
Ginger capsules	[30]	Application of Herbal Medicines for Obesity Treatment in the Polycystic Ovarian Syndrome Women	85 women	14 women treated with ginger capsules	Glucose, insulin, levels of both low and high-density lipoprotein levels ↓

↓—Decrease; ↑—Increase; IP—intraperitoneal; FSH—follicle stimulating hormone; LH—luteinizing hormone.

## Data Availability

Not applicable.

## Supplementary Materials

The following supporting information can be downloaded at: https://www.mdpi.com/article/10.3390/antiox13040392/s1, Table S1: Animal studies with antioxidant effect of ginger or cinnamon on reproductive system. Table S2: Clinical study with antioxidant effect ginger on respiratory system; Table S3: Animal and clinical studies with antioxidant effect ginger or cinnamon on urinary (excretory) system; Table S4: Animal studies with antioxidant effect ginger or cinnamon on nervous system; Table S5: Animal and clinical studies with antioxidant effect ginger or cinnamon on gastrointestinal system; Table S6: Animal and clinical studies with antioxidant effect ginger or cinnamon on endocrine system; Table S7: Animal studies with antioxidant effect ginger or cinnamon on cardiovascular system.
