# Supplementary material for: Exploring the Antioxidative Effects of Ginger and Cinnamon: A Comprehensive Review of Evidence and Molecular Mechanisms Involved in Polycystic Ovary Syndrome (PCOS) and Other Oxidative Stress-Related Disorders
Source: Antioxidants (Basel). 2024 Mar 25;13(4):392. doi: 10.3390/antiox13040392 (PMC11047656; doi:10.3390/antiox13040392)
Supplement: Supplementary file 1 [file antioxidants-13-00392-s001.zip › antioxidants-2848265-supplementary.docx]

**Table S1.** Animal studies with antioxidant effect of ginger or cinnamon on reproductive system.

|  |  | Reproductive system | | | | |
| --- | --- | --- | --- | --- | --- | --- |
|  | **Form of drug** | **Reference** | Sample size | Duration of study /doses | Parameters of interest |  |
|  | aqueous ginger  extract | [58] | 24 rats | aqueous ginger  extract (500 mg/kg BW/day) for 30 days | CAT↑  SOD↑  MDA↓  GSH↑ |  |
|  | ethanolic ginger extract | [59] | 20 rats | 100 mg/kg body weight of ethanolic *Zingiber officinale* extract (14 days); | SOD↑  GPx↑  CAT↑ |  |
|  | ginger powder | [60] | 24 rats | diet supplemented  with ginger roots at 3% for 1 month | CAT↑  SOD↑  GSH↑ |  |
|  | Ginger solution or cinnamon solution | [61] | 80 rats | 100mg/kg/rat  ginger and 75mg/kg cinnamon | SOD↑  MDA↓  GPx↑ |  |
|  | ginger rhizome extract | [62] | 28 rats | ginger rhizome extract (1 g/kg of  BW/d) by oral administration. | MDA↓ |  |

**Table S2 .** Clinical study with antioxidant effect ginger on respiratory system.

|  |  | Respiratory system | | | | |
| --- | --- | --- | --- | --- | --- | --- |
|  | **Form of drug** | **Authors** | Sample size | Duration of study /doses | Parameters of interest |  |
|  | Ginger powder | [63] | 69 patients | 3g Ginger powder form orally twice daily after meals for  30 days | MDA ↓ |  |

**Table S3.** Animal and clinical studies with antioxidant effect ginger or cinnamon on urinary (excretory) system.

|  |  | Urinary (excretory) system | | | | |
| --- | --- | --- | --- | --- | --- | --- |
|  | **Form of drug** | ***Authors*** | Sample size | Duration of study /doses | Parameters of interest |  |
|  | Ginger powder | [64] | 36 patients | 1000 mg of ginger as four capsules daily for 10 wk | MDA↓ |  |
|  | ginger extract | [65] | 30 rats | ginger extract (150 mg/kg BW) daily for 1 week  ginger extract (150 mg/kg BW)  daily for 3 weeks. | GSH↑  GPx↑  CAT↑ |  |
|  | Ginger powder | [66] | 24 rats | ginger powder as 5% of daily food intake  for 8 weeks | TBARS↓  MDA↓ |  |
|  | Cinnamon oil | [67] | 21 rats | cinnamon oil 5,  10 and 20mg/kg (dissolved in DMSO; i. p.), respectively, for 14  days | GSH↑  GPx↑  CAT↑ |  |
|  | Cinnamon oil | [68] | 36 rats | CO (50, 100 and 200 mg/kg) orally for 15 days | GSH↑  SOD↑  CAT↑  GPX↑ |  |
|  | *Cinnamon cassia* aqueous extract (CAE)* | [69] | 60 male rats | 200 mg CAE/ kg | MDA↓ |  |
|  | cinnamon extract | [70] | Male rats | Cinnamon extract  (200mg/kg b.w), for 6 weeks | MDA↓  SOD↑  CAT↑ |  |

**Table S4.** Animal studies with antioxidant effect ginger or cinnamon on nervous system.

|  |  | Nervous system | | | | |
| --- | --- | --- | --- | --- | --- | --- |
|  | **Form of drug** | **Authors** | Sample size | Duration of study /doses | Parameters of interest |  |
|  | Ginger juice | [71] | 84 rats | ginger juice (100 mg/kg bw) for  14 days  days | CAT↑  MDA↓  GSH↑ |  |
|  | ethanolic extract of ginger | [72] | 30 rats | ethanolic extract of ginger  with a dose of 200 mg/kg body weight via oral gavage for 30 days | SOD↑  CAT↑  GSH↑ |  |
|  | cinnamon oil | [73] | 36 rats | C 50, 100, and 200 mg/kg | GPx↑  SOD↑  CAT↑ |  |
|  | Cinnamonum verum) powder | [74] | 3 mice | Cinnamomum verum (100 mg/kg body wt/d) via gavage for 2 months | MDA↓  GSH↑ |  |
|  | cinnamon oil  ginger oil | [75] | 50 male rats | cinnamon oil in a dose of 400 mg/kg/bw  ginger oil (50 mg/kg/bw)  once a day for 4 weeks | MDA↓  GSH↑ |  |

**Table S5.** Animal and clinical studies with antioxidant effect ginger or cinnamon on gastrointestinal system.

|  |  | Gastrointestinal system | | | | | | | |
| --- | --- | --- | --- | --- | --- | --- | --- | --- | --- |
|  | **Form of drug** | **Authors** | Sample size | Duration of study /doses | Parameters of interest | | |  |  |
|  | Ginger powder | [76] | 64 patients | 12 weeks /  2000 mg/day | TAC-no change  MDA↓ | | |  |  |
|  | Aqueous solution of whole ginger | [77] | 48 rats | 160 mg/kg body weight for 50 days. | MDA↓  GSH↑  SOD↑  GPx↑ | | |  |  |
|  | 6-gingerol  capsule | [78] | 43 patients | two capsules of standardized 6-gingerol 5 mg or two capsules of placebo  twice daily starting from 3 days prior to receiving their first  cycle of chemotherapy and continued taking daily through  the fourth cycle of chemotherapy. | SOD↑  CAT↑  GPx↑ | | |  |  |
|  | cinnamon polyphenol extract | [79] | 28 wistar rats | cinnamon polyphenol extract [100mg/kg b.wt.  dissolved in 5% dimethyl sulfoxide (DMSO)] daily by oral  gavage in olive oil (1ml/kg b.wt./day) | MDA↓  TAC↑  SOD↑  CAT↑  GSH↑  GPx↑ | | |  |  |
|  | Aqueous cinnamon extract | [80] | 32 male wistar rats | acrylamide- intoxicated rats treated with CE 250 mg/kg/day or CE 500 mg/kg/day | TAC↑  MDA↓ | | |  |  |
|  | Cinnamon oil | [81] | 36 rats | CO at 50 mg/kg b.wt.,  CO at 100 mg/ kg b.wt.  , CO at 200 mg/ | MDA↓  GSH↑  SOD↑  CAT↑  GPx↑ | | |  |  |
|  | *cinnamon extract* | [82] | 40 rats | 100 mg/kg of  aqueous or ethanolic extracts orally for 7 days | MDA↓  SOD↑  CAT↑ | | |  |  |
|  | *cinnamon extract* | [83] | 48 male albino rats | orally to rats at dosage of 20mg/day/rat.  for 3 and 6 weeks | MDA↓ | | |  |  |
|  | Cinnamon decoction | [84] | 24 rats | Cinnamon decoction 10% (3 ml/kg, p.o) for  7 days prior to stress.  Or Cinnamon decoction 10% (6 ml/kg, p.o) for  7 days prior to stress | SOD↑  CAT↑ | | |  |  |
|  | Cinnamon decoction | [85] | 24 rats | cinnamon decoction  10%, 3 and 6 ml/kg, p.o, for 7 days | NO↓  TBARS↓  MDA↓  GSH↑  CAT↑ | | |  |  |
|  | Cinnamon extract | [86] | 42 rats | cinnamon extract, 200 mg/kg | MDA↓  H_2_O_2↓_  NO↓ | | |  |  |
|  |  |  |  | | |  |  | |  |

**Table S6.** Animal and clinical studies with antioxidant effect ginger or cinnamon on endocrine system.

|  |  | Endocrine system | | | | |
| --- | --- | --- | --- | --- | --- | --- |
|  | **Form of drug** | ***Authors*** | Sample size | Duration of study /doses | Parameters of interest |  |
|  | Ginger rhizome powder | [87] | 32 patients | 1 g ginger/day for 10 weeks | TAC↑  MDA↓ |  |
|  | Aqueous extract of cinnamon | [88] | 22 patinets | placebo or 250 mg of a dried aqueous extract of  cinnamon (Cinnulin PF) two times per day for 12 weeks | MDA↓  SOD↑  GPx↑ |  |
|  | Cinnamon powder capsules | [89] | 49 patients | cinnamon capsules (500 mg) or placebo capsules after  meal three times a day (as suggested in the leaflet),  everyday for a total of 60 days. | MDA↓  TAC↑ |  |
|  | Ginger powder  Cinnamon powder | [90] | 60 healthy women | 3 grams of oral dietary ginger powder (*n* = 15)  or dietary cinnamon powder (*n* = 15) | MDA↓ |  |
|  | Ginger powder capsules | [91] | 41 patients | received 2 g/day of ginger powder supplement or lactose as placebo for 12 weeks | MDA↓ |  |
|  | Ginger powder | [92] | 42 rats | Ginger powder and mixed  with diet to get 1% and 2% supplementation and dietary was given to rats for 30 days | SOD↑  CAT↑  GPx↑ |  |
|  | Ginger powder | [93] | 40 rats | 1% ginger powder mixed into food | TBARS↓  SOD↑  CAT↑ |  |
|  | Ginger extract | [94] | 60 rats | distilled water, honey  (2 g/kg body weight), ginger (60mg/kg body weight), and honey + ginger | SOD↑  CAT↑  GPx↑ |  |
|  | Aqueous ginger extract (AGE)  ethanol ginger extract (EGE). | [95] | 36 rats | AGE 250 mg/kg body weight;  AGE 500 mg/kg body weight  EGE 250 mg/kg body weight EGE 500 mg/kg body weight  for 6 weeks | MDA↓  SOD↑  CAT↑  GSH↑ |  |
|  | *Cinnamon zeylanicum*  Powder dissolved in distilled water | [96] | 40 wistar rats | Cinnamon  75mg/kg daily for 30 days | MDA↓  GPx↑  SOD↑ |  |
|  | Cinnamon  extract | [97] | 60 male Wistar rats | cinnamon extract at dose of 100 mg/kg body weight daily for 2 months.  orally freshly prepared cinnamon extract at dose of 200 mg/kg body weight daily for 2 months. | MDA↓  SOD↑  GSH↑ |  |
|  | *Cinnamon cassia* bark extract (CCBE) | [98] | 20 rats | 0.3 grams and 0.6 grams of CCBE | SOD↑  GPx↑ |  |

**Table S7.** Animal studies with antioxidant effect ginger or cinnamon on cardiovascular system.

|  |  | Cardiovascular system | | | | |
| --- | --- | --- | --- | --- | --- | --- |
|  | **Form of drug** | **Authors** | Sample size | Duration of study /doses | Parameters of interest |  |
|  | *Cinnamomum zeylanicum*  Extract (CZ) | [99] | 32 rats | (CZ) extract was given in three different doses of 50, 100, and 200 mg/kg/day for 14 days | SOD↑  GPx↑  CAT↑ |  |
|  | *Cinnamomum zeylanicum*  Extract (CZ) | [100] | 50 rats | Cinnamon extract [200 mg/kg b.wt] | TAC↑  GPx↑  MDA↓  SOD↑ |  |
